# A critical base pair in k-turns that confers folding characteristics and correlates with biological function

**DOI:** 10.1038/ncomms6127

**Published:** 2014-10-29

**Authors:** Scott A. McPhee, Lin Huang, David M. J. Lilley

**Affiliations:** 1 Cancer Research UK Nucleic Acid Structure Research Group, MSI/WTB Complex, The University of Dundee, Dow Street, Dundee DD1 5EH, UK

## Abstract

Kink turns (k-turns) are widespread elements in RNA that mediate tertiary contacts by kinking the helical axis. We have found that the ability of k-turns to undergo ion-induced folding is conferred by a single base pair that follows the conserved A·G pairs, that is, the 3b·3n position. A Watson–Crick pair leads to an inability to fold in metal ions alone, while 3n=G or 3b=C (but not both) permits folding. Crystallographic study reveals two hydrated metal ions coordinated to O6 of G3n and G2n of Kt-7. Removal of either atom impairs Mg^2+^-induced folding in solution. While SAM-I riboswitches have 3b·3n sequences that would predispose them to ion-induced folding, U4 snRNA are strongly biased to an inability to such folding. Thus riboswitch sequences allow folding to occur independently of protein binding, while U4 should remain unfolded until bound by protein. The empirical rules deduced for k-turn folding have strong predictive value.

## Abstract (web summary)

The k-turn is a widespread RNA element that adopts a kinked structure that mediates tertiary contacts and frequently binds specific proteins. Here, McPhee *et al*. show that the ability of a given k-turn to fold in the presence of metal ions alone—or to otherwise require protein binding—is attributable to a specific base pair.

## Graphical abstract

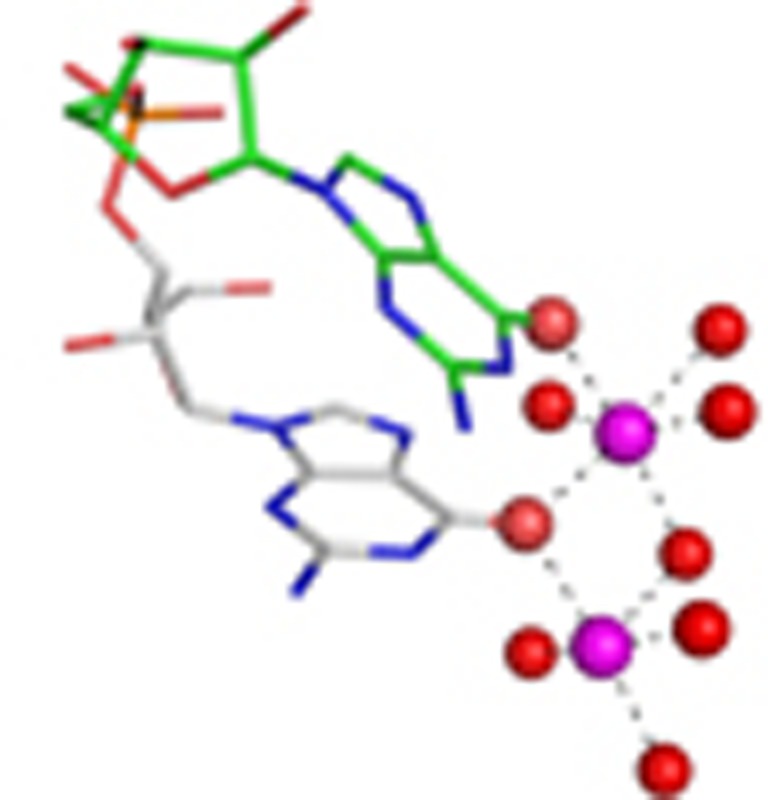

Kink turns (k-turns) are ubiquitous sequences that generate a tight kink within an RNA helix^1^, mediating tertiary interactions in the folding of large assemblies such as the ribosome, and often serving as the target for specific binding proteins. Because of this, k-turns have a key role in the assembly of ribosomes, the spliceosome^2^ and box C/D^3^ and H/ACA^4^,^5^ snoRNPs, as well as seven distinct riboswitch species^6^. The standard k-turn comprises a duplex interrupted by a three-nucleotide bulge followed by G·A and A·G base pairs (Fig. 1), and the adenine nucleobases make key cross-strand hydrogen bonds that stabilize the kinked conformation^7^,^8^,^9^.

k-turn-containing RNA exists in a two-state equilibrium between the kinked conformation and a relatively extended structure, and is strongly biased towards the extended structure in the absence of some specific process promoting folding^10^. Several factors can drive the equilibrium towards the kinked structure of the k-turn. These include tertiary contacts^11^ and protein binding^12^,^13^,^14^. The L7Ae family^15^ is a particularly important class of proteins that selectively bind k-turns, that includes the human 15.5 kDa protein. L7Ae-related proteins are bound to k-turns in the ribosome^16^, U4 snRNA^17^ and box C/D^18^,^19^. Metal ions are a third factor—some but not all, k-turns will fold upon addition of metal ions. Both divalent and monovalent ions can induce k-turn folding, but much higher concentrations of the latter are required. Half-complete folding of Kt-7 occurs with [Mg^2+^]_1/2_=90 μM or [Na^+^]_1/2_=30 mM (ref. 10).

However k-turns differ markedly in their ability to fold in response to metal ions. Some will fold intrinsically in the presence of physiological concentrations of metal ions, while others require stabilization by other means, such as the binding of proteins. These contrasting folding properties must be very important in the ordered assembly and function of their RNA species. In this work we have set out to discover the molecular basis of these differences. We have discovered the key determinant of ability to undergo ion-induced folding resides in a single base pair that follows the conserved A·G pairs (3b·3n). The most readily folded sequence has A·G in this position, and we find that the O6 of the guanine directly coordinates metal ions. Analysis of many sequences of the k-turns of the SAM-I riboswitch and U4 snRNA reveals a strong correlation between the folding ability conferred by their sequence, and their biological function. The deduced sequence rules have strong predictive value, and can be applied to many natural RNA sequences such as those of the ribosome.

## Results

### Ion-induced folding determined by a key sequence element

**Figure 1:**
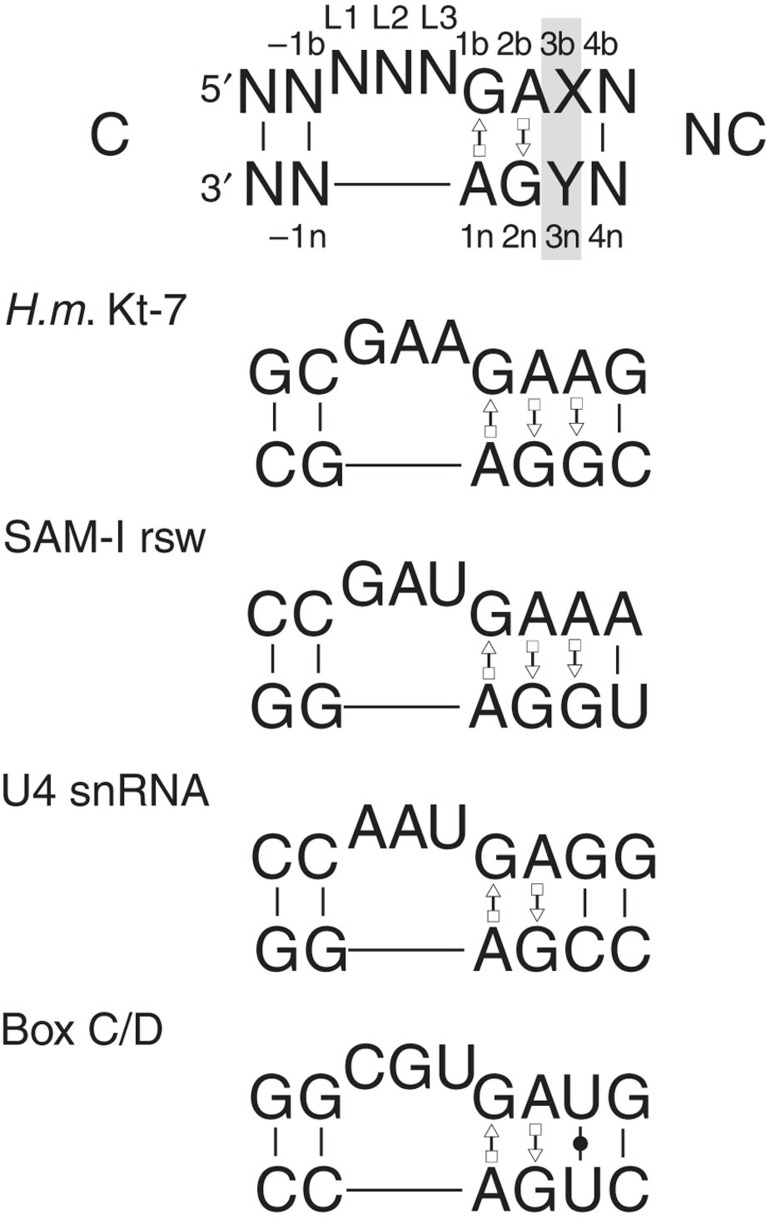
The sequences of some standard k-turns. The top sequence shows a standard k-turn with the standard nomenclature for nucleotide positions indicated^8^ and the 3b·3n position highlighted. Below are shown the sequences of the k-turns of *H. marismortui* ribosomal Kt-7, the SAM-I riboswitch, human U4 snRNA and box C/D snoRNA.

The extensively-studied Kt-7 of the *Haloarcula marismortui* ribosome, and the k-turn of the SAM-I riboswitch, both fold into the characteristic kinked structure on addition of metal ions alone (Supplementary Figs 1 and 2; refs 10,11). However, in marked contrast the k-turns of the archaeal box C/D (Supplementary Fig. 3) and the human U4 snRNA (Supplementary Fig. 4) do not fold upon addition of metal ions. Both box C/D and U4 k-turns fold on binding of L7Ae protein, and indeed co-crystal structures of both show that these k-turns are folded^2^,^3^, so each is intrinsically capable of adopting the k-turn conformation, yet metal ions alone fail to achieve folding. These biologically important k-turns divide into two classes on the basis of their ability to be folded by metal ions, evidently a result of their sequence. As the G·A and A·G pairs are strongly conserved at the 1b·1n and 2b·2n positions (the nomenclature^8^ is shown in Fig. 1), the important difference must lie elsewhere, and our suspicion turned to the 3b·3n position that follows the conserved G·A pairs.

**Figure 2:**
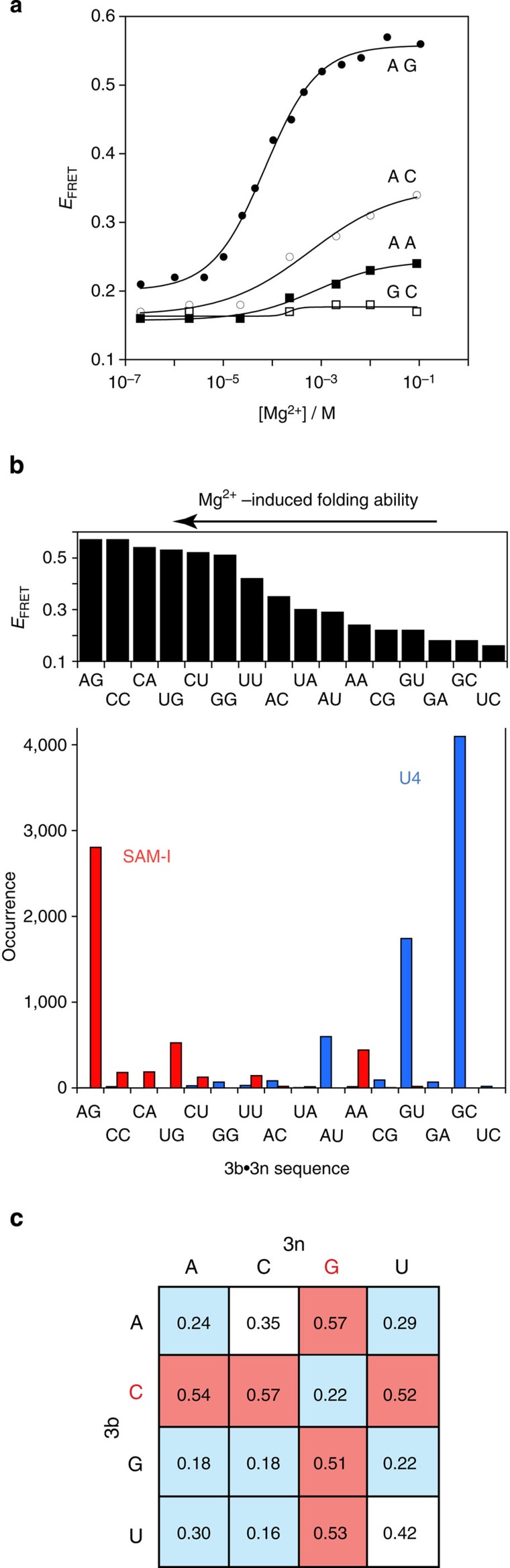
The ion-induced folding of k-turns as a function of sequence, and a correlation with biological function. (**a**) Plot of *E*_FRET_ (measured in the ensemble) as a function of Mg^2+^ ion concentration for Kt-7 (3b·3n=A·G) and three representative 3b·3n variants of contrasting folding ability. The data (points) have been fitted (lines) to a two-state model for ion-induced folding. (**b**) Histograms of *E*_FRET_ versus 3b·3n sequence (upper) and distribution of SAM and U4 3b·3n sequences (lower) in natural sequences found in the Rfam database^20^ ranked (L to R) by the ability to fold in Mg^2+^ ions. (**c**) An array showing *E*_FRET_ as function of 3b (vertical) and 3n (horizontal) sequence for all Kt-7 variants. Sequences that are readily folded by Mg^2+^ ions (Δ*E*_FRET_≥0.5) are shaded red, while those poorly folded (Δ*E*_FRET_≤0.3) are shaded blue. The ascending diagonal contains the Watson–Crick pairs.

We took a short RNA duplex with a central Kt-7 sequence and fluorophores at both 5′ termini, enabling us to follow folding into the kinked conformation by the increase in FRET efficiency (*E*_FRET_) as the end-to-end distance shortens. The experiment was performed in a background of 90 mM Tris-borate (pH 8.3), with Mg^2+^ titrated as the only cation present. For the natural Kt-7 sequence *E*_FRET_ increased from ~0.2 to 0.56 on addition of Mg^2+^ ions, with a value of [Mg^2+^]_1/2_=70 μM. The analysis was repeated for the 15 species in which the 3b·3n position was replaced by each combination of the four nucleotides (Fig. 2a, Supplementary Table 1). A range of folding abilities were found from full folding (for example, natural Kt-7; 3b·3n=A·G) to those exhibiting a complete inability to fold under these conditions (for example, 3b·3n=G-C). Yet even the 3b·3n=G-C sequence underwent folding upon addition of L7Ae protein, so it is not intrinsically unable to adopt the k-turn structure.

### 3b·3n sequences correlate with biological function

Thus the 3b·3n sequence acts as a key discriminator, conferring ion-dependent folding properties. Is this reflected in the distribution of k-turn sequences as a function of biological role? We examined the distribution of 3b·3n sequences in two important functional RNA species, comparing several thousand SAM-I and U4 k-turn sequences downloaded from the Rfam database^20^. These two were chosen because of their contrasting environments. The SAM-I k-turn mediates a key tertiary contact^6^,^11^ to create a ligand-binding pocket in a riboswitch not known to bind a protein, while the U4 snRNA k-turn binds the 15.5 kDa protein during spliceosome assembly^17^. The results are plotted as a histogram in Fig. 2b, showing the occurrence of the 3b·3n sequences for the two species ranked horizontally by the folding ability of Kt-7 with the same 3b·3n sequence. It is apparent that the two species cluster at opposite ends of the folding spectrum. The SAM-I k-turn sequences are strongly biased towards an ability to fold in Mg^2+^ ions, with 60% having 3b·3n=A·G, which is the best folding sequence, and just 0.1% being C-G or G-C. By contrast, 97% of the U4 k-turn sequences are predicted to be unable to fold in Mg^2+^ ions, with a very strong bias to 3b·3n=G-C or G-U, and less than 0.03% being A·G. Interestingly, modification of the human U4 k-turn sequence by conversion of 3b·3n from G-C to A·G conferred an ability to fold in response to addition of Mg^2+^ ions (Supplementary Fig. 4).

### Empirical sequence rules for ion-induced folding

Examination of the 3b·3n sequences displayed in array form and scored by folding ability (Fig. 2c) reveals some interesting patterns. First, Watson–Crick pairs (Fig. 2c—ascending diagonal) plus GU are all poor folders, with G-C and C-G especially bad. By contrast, the presence of 3n=G (Fig. 2c—third column) or 3b=C (Fig. 2c—second row) associates with ability to fold in Mg^2+^ ions. However, since 3b·3n=C-G is unfolded in metal ions, the first rule takes precedent over the second. The two best-folding k-turns both have G at the 3n position, and 95% of SAM-I k-turns have G at 3n. Moreover, the k-turns of the glycine^21^, lysine^22^ and cobalamine^23^ riboswitches also have 3n=G.

### A structural explanation of the 3n=G rule

Systematic investigation of the ion-induced folding of Kt-7 shows that the most readily folded sequences are those with either 3n=G or 3b=C. There are no high-resolution crystal structures available for 3b=C k-turns, and at the present time we cannot rationalize this effect. However, we can provide a molecular explanation for the 3n=G behaviour.

**Figure 3:**
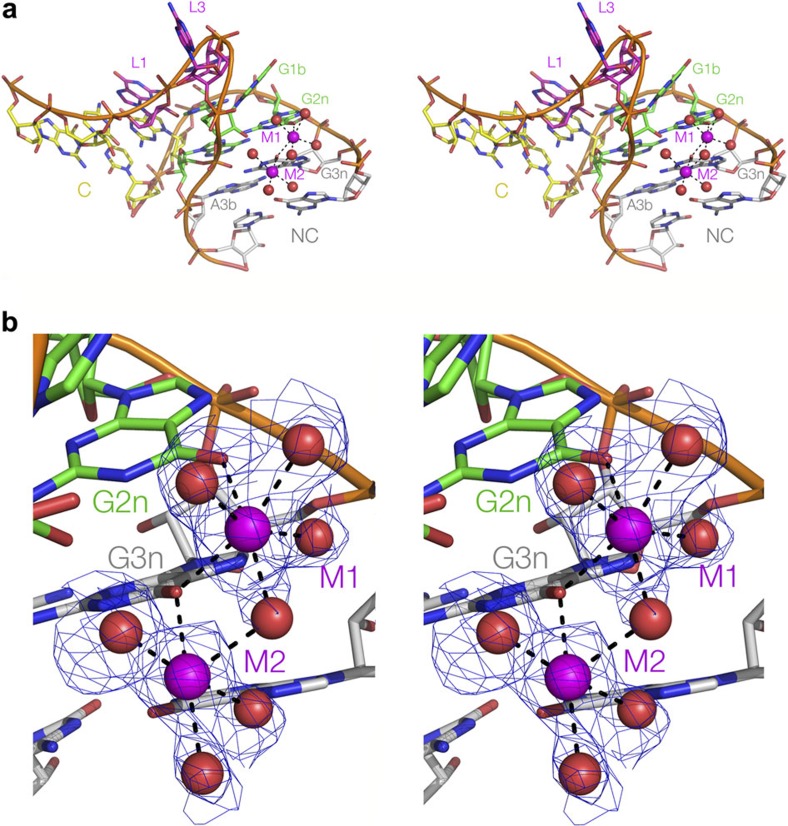
A structural basis for the differential ability of k-turn sequences to undergo ion-induced folding. (**a**,**b**) Crystal structure of Kt-7 reveals two Mg^2+^ ions bound in the major groove of the NC helix. The structure is shown as parallel eye stereographic images. (**a**) An overall view of the k-turn, with bound ions. (**b**) Closer view of the bound ions. The electron density for the metal and directly bound water is taken from the **F**_o_−**F**_c_ omit map contoured at 2σ. Further maps are shown in Supplementary Figure 5. Both ions have exchanged inner-sphere water ligands to bond to guanine O6 atoms; M1 is directly bonded to O6 of both G2n and G3n, while the latter is directly bonded to both ions.

We have previously presented a crystal structure of *H. marismortui* Kt-7 as a protein-free duplex at 2.3 Å resolution^24^. We subsequently obtained crystals diffracting to 2.0 Å (Table 1), whereupon we observed two hydrated metal ions bound in the major groove of the NC helix adjacent to G2n and G3n (Fig. 3a). The electron density for the inner coordination sphere of water molecules is very clear, and both metal ions have octahedral symmetry. Thus they are most probably Mg^2+^ ions, although we cannot exclude the possibility that they are Na^+^ ions on the basis of the metal-O distances. Ion M1 has exchanged two adjacent inner-sphere water molecules with G2n and G3n O6 atoms, while G3n O6 makes an inner-sphere contact with both ions (Fig. 3b, Supplementary Fig. 5).

### Removal of O6 from G2n or G3n impairs ion-induced folding

**Figure 4:**
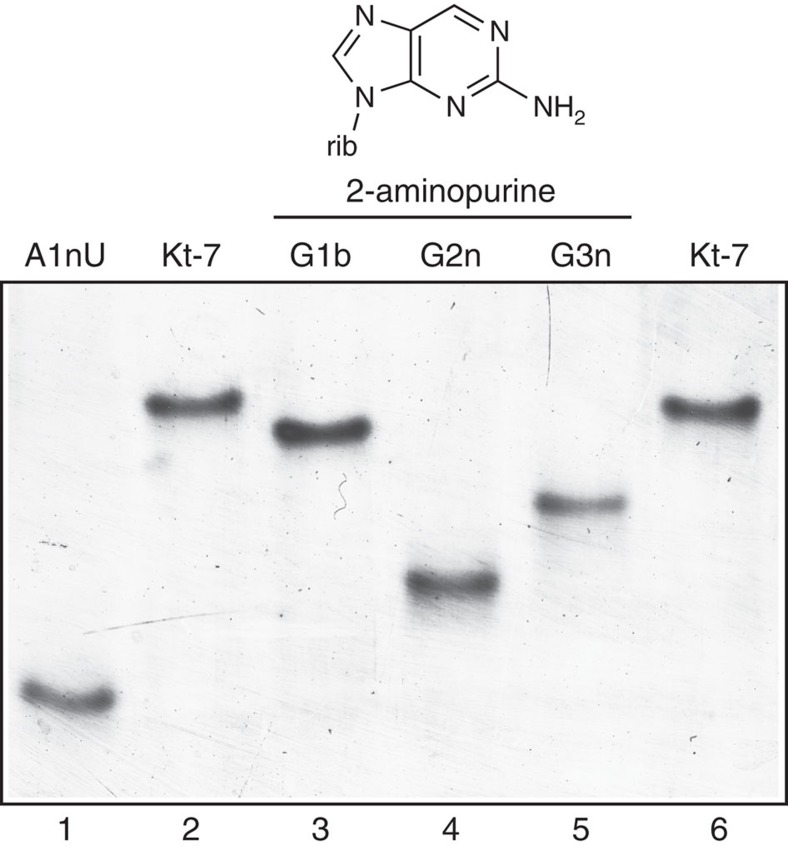
Removal of the O6 atoms from G2n and G3n impairs ion-induced folding of Kt-7. The scheme at the top shows the structure of 2-aminopurine, showing that it differs from guanine by removal of O6. A 65 bp duplex with a central k-turn-containing RNA section was electrophoresed in 15% polyacrylamide in the presence of 2 mM Mg^2+^ ions. Folding of the unmodified species (tracks 2 and 6) results in a marked retardation compared with the A1nU variant (track 1). Substitution of G2n (track 4) or G3n (track 5) by 2-aminopurine lead to a marked impairment of folding (that is, increased mobility), whereas the same substitution at G1b (track 3) had a rather smaller effect.

Having observed the two ions bound to G3n and G2n in the crystal, we then sought to test the importance of these interactions in the folding of Kt-7 in solution. This was examined by atomic mutagenesis whereby the participating O6 atoms were selectively removed by individual substitution of guanine by 2-aminopurine. Folding was analysed using a gel electrophoretic method^10^. A 65 bp RNA duplex with a central k-turn-containing RNA section was electrophoresed in 15% polyacrylamide in the presence of 90 mM Tris-borate (pH 8.3), 2 mM Mg^2+^. The folded structure of the unmodified k-turn results in pronounced electrophoretic retardation (Fig. 4). However, removal of either G2n or G3n O6 atoms significantly impaired the ability to fold on addition of Mg^2+^ ions, that is, resulted in less retarded electrophoretic mobility, whereas the corresponding modification of G1b had a minor effect. This provides a direct connection between the metal ions observed to bind to G2n and G3n O6 atoms by crystallography, and the ability of the k-turn to fold in response to the presence of Mg^2+^ ions. Thus binding of the divalent metal ions to guanine O6 at the 2n and 3n positions is the key determinant allowing the Kt-7 k-turn to fold unassisted by protein binding.

## Discussion

While all k-turns can be folded by protein binding and/or the formation of tertiary contacts, not all will fold spontaneously in the presence of metal ions, and we have found that a major determinant of this behaviour resides in the 3b·3n sequence. From a systematic analysis of 3b·3n sequence variants of Kt-7, we have formulated a set of rules that have predictive value; application of these can convert the U4 k-turn from non-ion folding into one that is fully folded, in the presence of Mg^2+^ ions for example. One of the rules is that 3n=G, and a high-resolution structure of Kt-7 as a free duplex RNA provides an explanation. Two hydrated, octahedrally-coordinated metal ions are directly bound to the O6 atoms of G2n and 3n. These are probably Mg^2+^ ions, although they could conceivably be Na^+^ ions, but since both ions can induce folding of Kt-7 then probably either can coordinate at this position. The binding can be directly connected with folding in solution, since selective removal of the O6 atoms leads to impairment of folding in the presence of Mg^2+^ as the only cation.

Analysis of the sequences of the k-turns of SAM-I and U4 snRNA shows a striking difference in the 3b·3n sequences of the two species. The k-turn sequences of the SAM-I riboswitch have been selected for predisposition to fold in metal ions alone. There is no protein that is known to bind to riboswitches *in vivo*, so this property is probably essential to permit folding and thereby generate a functional riboswitch. By contrast, the U4 k-turn binds the 15.5 kDa protein *in vivo*, and evidently U4 sequences have been selected for their inability to fold in metal ions alone. The U4 k-turn will be unfolded until the protein is bound, and can therefore only function as an RNA-protein complex to generate the U4/U6 snRNA complex in the spliceosome cycle. In the absence of protein binding, the extended form of the k-turn should be more flexible and this may be required to permit the formation of other interactions during the biogenesis of this complex and dynamic assembly. This analysis reveals a strong correlation between the folding properties of the isolated k-turn and their likely role in the cellular macromolecule. It emphasizes the key role of the 3b·3n sequence in the biological function of these k-turns.

We can apply our deduced folding sequence rules to the k-turns of the *H. marismortui* 50S ribosomal subunit. Kt-7, Kt-46, Kt-58 and Kt-78 (ref. 16), plus the J4,5 k-junction^25^ all have 3b·3n=A·G, and are therefore likely to fold unaided by protein. Interestingly, analysis of 2,716 bacterial Kt-7 sequences shows that while 3b·3n=A·G is relatively uncommon, 99.9% have either 3n=G or 3b=C. These k-turns all mediate tertiary interactions, and we envision that during rRNA folding this will assist the formation of long-range contacts before the structure becomes fixed by the binding of specific proteins. By contrast, Kt-15 is a complex k-turn with 3b·3n=C-G, and it does not undergo folding by addition of Mg^2+^ ions alone (unpublished data). In the ribosome it is bound by L7Ae. *In vitro* L7Ae binds k-turns with pM affinity^13^. The folding of Kt-15 and its tertiary contacts should therefore occur later than those not requiring protein binding, all of which will contribute to an ordered process for the folding of the ribosome.

In summary, we have found a strong correlation between the folding properties conferred by the 3b·3n sequence and the biological role of specific k-turns. The deduced sequence rules have predictive value and can be applied to new k-turn sequences.

## Methods

### RNA synthesis

Ribooligonucleotides were synthesized using *t*-BDMS phosphoramidite chemistry^26^. Fluorescein (Link Technologies) and Cy3 (GE Healthcare) were attached at 5′ termini as phosphoramidites during synthesis as required. Oligoribonucleotides were deprotected in 25% ethanol/ammonia solution at 20 °C for 3 h, and evaporated to dryness. They were redissolved in 100 μl dimethyl sulfoxide to which was added 125 μl 1 M triethylamine trihydrofluoride (Sigma-Aldrich) and incubated at 65 °C for 2.5 h to remove *t*-BDMS protecting groups. All oligonucleotides were purified by gel electrophoresis in polyacrylamide in the presence of 7 M urea. The full-length RNA product was visualized by ultraviolet shadowing. The band was excised and electroeluted using an Elutrap (Whatman) into 45 mM Tris-borate (pH 8.5), 5 mM EDTA buffer for 8 h at 200 V at 4 °C. The RNA was precipitated with ethanol, washed once with 70% ethanol and suspended in water.

Oligoribonucleotides containing 2-aminopurine (Glen Research) were deprotected using a 1:1 solution of 35% aqueous ammonia (Fisher Scientific) and 40% aqueous methylamine (Sigma-Aldrich) for 30 min. at 65 °C.

Fluorophore-labelled and 2-aminopurine-containing oligoribonucleotides were subjected to further purification by reversed-phase HPLC on a C18 column (ACE 10–300, Advanced Chromatography Technologies), using an acetonitrile gradient with an aqueous phase of 100 mM triethylammonium acetate (pH 7.0).

Duplex species were prepared by mixing equimolar quantities of the appropriate oligoribonucleotides and annealing them in 50 mM Tris–HCl (pH 7.5), by slow cooling from 90 to 4 °C. They were purified by electrophoresis in 12% polyacrylamide under nondenaturing conditions and recovered by electroelution, followed by ethanol precipitation.

### Expression and purification of *A. fulgidus* L7Ae

The gene encoding full-length *Archaeoglobus fulgidus* L7Ae was cloned into a modified pET-Duet1 plasmid (Novagen)^28^ using the *Hin*dIII and *Eco*RI sites. The L7Ae gene was fused upstream of a hexahistidine-encoding sequence with a PreScission-cleavable linker. The hexahistidine-L7Ae fusion protein was expressed in *Escherichia coli* BL21-Gold (DE3) pLysS cells (Stratagene) induced with 0.2 mM IPTG at 20 °C for 12 h.

Harvested cells were resuspended in 20 mM Tris–HCl, (pH 8.0), 500 mM NaCl, 10 mM imidazole, 1 mM phenylmethylsulfonyl fluoride (buffer A) and lysed by sonication. The protein suspension was heated at 85 °C for 20 min in the presence of 10 mM MgCl_2_ to denature endogenous protein and this was removed by centrifugation at 18,000 r.p.m. for 30 min at 4 °C. L7Ae was loaded onto a HisTrap column (GE Healthcare), washed with 25 mM imidazole in buffer A, and the protein was eluted with 500 mM imidazole in buffer A. The six-His tag was cleaved from L7Ae by PreScission protease in 20 mM HEPES-Na (pH 7.6), 100 mM NaCl, 0.5 mM EDTA (buffer C) at 4 °C for 16 h. L7Ae was applied to a heparin column (GE Healthcare) and eluted at 250 mM NaCl in a gradient from 50 to 2,000 mM NaCl in 20 mM HEPES-Na (pH 7.6). The protein was further purified using a Superdex 200 gel filtration column in a buffer containing 5 mM Tris-HCl (pH 8.0), 100 mM NaCl.

The protein concentration was measured by absorbance at 280 nm using a molar extinction coefficient of 5,240 M^−1^ cm^−1^ for L7Ae. The protein was concentrated to 20 mg ml^−1^ in buffer containing 5 mM Tris-HCl (pH 8.0), 100 mM NaCl and stored at −20 °C as aliquots.

### FRET analysis of k-turn folding

FRET efficiency was measured from a series RNA duplex species terminally 5′-labelled with fluorescein and Cy3, containing central k-turn sequences and variants.

Absorption spectra were measured in 90 mM Tris-borate (pH 8.3) in 2 μl volumes using a Thermo Scientific NanoDrop 2000c spectrophotometer. Spectra were deconvoluted using a corresponding RNA species labelled only with Cy3, and fluorophore absorption ratios calculated using a MATLAB program. Fluorescence spectra were recorded in 90 mM Tris-borate (pH 8.3) at 4 °C using an SLM-Aminco 8,100 fluorimeter. Spectra were corrected for lamp fluctuations and instrumental variations, and polarization artifacts were avoided by setting excitation and emission polarizers crossed at 54.7°. Values of FRET efficiency (*E*_FRET_) were measured using the acceptor normalization method^29^ implemented in MATLAB. *E*_FRET_ as a function of Mg^2+^ ion concentration was analysed on the basis of a model in which the fraction of folded molecules corresponds to a simple two-state model for ion-induced folding, that is,


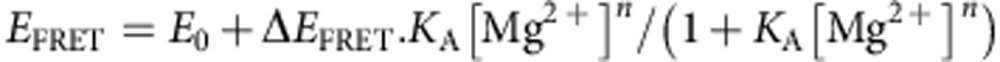


where *E*_0_ is the FRET efficiency of the RNA in the absence of added metal ions, Δ*E*_FRET_ is the increase in FRET efficiency at saturating metal ion concentration, [Mg^2+^] is the prevailing Mg^2+^ ion concentration, *K*_A_ is the apparent association constant for metal ion binding and *n* is a Hill coefficient. Data were fitted to this equation by nonlinear regression. The metal ion concentration at which the transition is half complete is given by [Mg^2+^]_1/2_=(1/*K*_A_)^1/*n*^.

The same RNA oligonucleotides as used in the Mg^2+^-induced folding were used for the L7Ae binding experiments, and FRET was measured and analysed using the same approach. L7Ae was added from a stock solution to a solution of 2 nM solution of RNA.

### Gel electrophoretic analysis of k-turn folding

RNA species were electrophoresed in 13% polyacrylamide (29:1, acrylamide:bis) gels in 90 mM Tris.borate (pH 8.3) plus 2 mM Mg^2+^ ions. Electrophoresis was performed at 120 V at 4 °C for at least 72 h, with recirculation of the buffer at >1 litre h^−1^. Gels were stained using SYBR Gold (Life Technologies), washed in MilliQ water and visualized on a Typhoon FLA 9500 (GE Healthcare).

The sequences used for the electrophoretic experiments were (written 5′ to 3′):

Kt-7 upper strand:

5′- CGCAAGCGACAGGAACCTCGCCAGUCAGUGGCGAAGAACCAU GUCAGGGGACTGTCAAGTTGAACAGG -3′

Kt-7 lower strand:

5′- CCTGTTCAACTTGACAGTCCCCUGACAUGGGGAGCCACUGA CUGGCGAGGTTCCTGTCGCTTGCG -3′

The DNA sections of these oligonucleotides are shown underlined. Modified nucleotides were introduced into the RNA sections as required.

### Crystal structure determination and refinement

The crystallized construct had the sequence (written 5′ to 3′):

5′- GGCGAAGAACCGGGGAGCC -3′

This self-complementary sequence forms the structure shown in Supplementary Fig. 6, containing two Kt-7 motifs.

A solution of 1 mM RNA in 5 mM Tris–HCl (pH 8.0) and 100 mM NaCl was heated to 95 °C for 1 min. The solution was slow cooled to 20 °C and MgCl_2_ was added to a final concentration of 10 mM. The hanging-drop vapour diffusion method was used for crystallization. A volume of 1.0 μl of RNA was mixed 1:1 with well solution comprising 3.5 M Na formate, 0.1 M Na acetate (pH 4.6) at 20 °C. Crystals (approximate dimensions 150 × 20 × 20 μm^3^) with space group P6_3_22 grew in a few days. Crystals were briefly washed in well solution supplemented with 30% glycerol. The crystals were flash frozen by mounting in nylon loops and plunging into liquid nitrogen. A 2.0 Å resolution data set was collected on beamline I03 of the Diamond Light Source (Harwell, UK). The resolution cutoff for the data was determined by examining both CC1/2 and difference map of the magnesium ions, as described previously^30^,^31^. The structure was determined by molecular replacement. *H. marismortui* Kt-7 (PDB 4C40) was used as the search model using the program PHASER^32^. The remaining ligands and waters were added to the model on the basis of inspection of electron density difference maps.

Structural models were built in Coot^33^ and RCrane^34^. The structure was refined with Refmac5 (ref. 35) from the CCP4 suite of programs^36^ and Phenix refine^37^. Model geometry and the fit to electron-density maps were monitored with MOLPROBITY^38^ and the validation tools in COOT.

## Author contributions

S.A.M. performed spectroscopy, biochemistry, bioinformatics and synthesis; L.H. performed crystallography; S.A.M., L.H. and D.M.J.L. devised experiments, analysed the data and wrote the paper.

## Additional information

**How to cite this article**: McPhee, S. A. *et al*. A critical base pair in k-turns that confers folding characteristics and correlates with biological function. *Nat. Commun.* 5:5127 doi: 10.1038/ncomms6127 (2014).

**Accession codes**: Atomic coordinates and structure factor amplitudes have been deposited with the PDB with accession code 4CS1.

## Supplementary Material

Supplementary Information Supplementary Figures 1-6 and Supplementary Tables 1

## Figures and Tables

**Table 1:** Data collection and refinement statistics.

Macromolecules	Kt-7
*Data collection*	
Space group	P6_3_22
Cell dimensions	
*a, b, c* (Å)	70.08, 70.08, 47.52
α, β, γ (°)	90.00, 90.00, 120.00
Resolution (Å)	37.41-2.00 (2.05-2.00)*
*R*_sym_ or *R*_merge_	6.5 (132.8)
I/σI	14.82 (1.4)
CC(1/2)	0.999 (0.501)
Completeness (%)	98.32 (99.59)
Redundancy	6.3 (6.4)
	
*Refinement*	
Resolution (Å)	37.42-2.00 (2.52-2.00)
No. of reflections	4916
*R*_work_/*R*_free_	0.184/0.234 (0.259/0.291)
No. of atoms	
RNA	417
Ligand/ion	2
Water	23
*B*-factors	
RNA	47.6
Ligand/ion	33.7
Water	44.6
r.m.s deviations	
Bond lengths (Å)	0.004
Bond angles	0.81

^*^Highest resolution shell is shown in parenthesis.
