# Supplementary material for: A critical base pair in k-turns that confers folding characteristics and correlates with biological function
Source: Nat Commun. 2014 Oct 29;5:5127. doi: 10.1038/ncomms6127 (PMC4382518; doi:10.1038/ncomms6127)
Supplement: Supplementary Information — Supplementary Figures 1-6 and Supplementary Tables 1 [file ncomms6127-s1.pdf]

SUPPLEMENTARY INFORMATION

Supplementary Data

Figures 1 through 4 show the ion-induced folding of k-turns studied by steady-state FRET. FRET efficiency (E_{FRET}) was measured using a duplex RNA containing a central k-turn motif 5-terminally labelled with fluorescein and Cy-3 fluorophores. In each case ΔE_{FRET} is plotted as a function of Mg^{2+} ion concentration. The data have been fitted to a two-state model for ion-induced folding. The RNA sequences used are shown above the plots, with the 3b•3n sequence highlighted red.

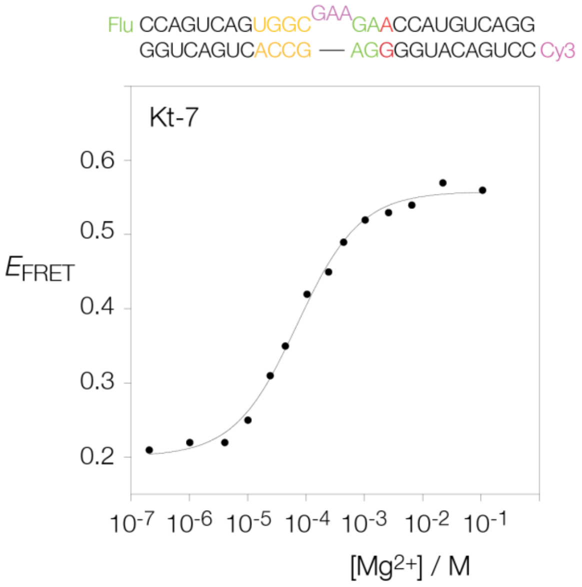

Supplementary Figure 1. The folding of natural sequence Kt-7 from *Haloarcula marismortui*. The k-turn folds fully in response to the addition of Mg^{2+} ions. The experimental data are shown by the filled circles, and the line is the fit to the data.

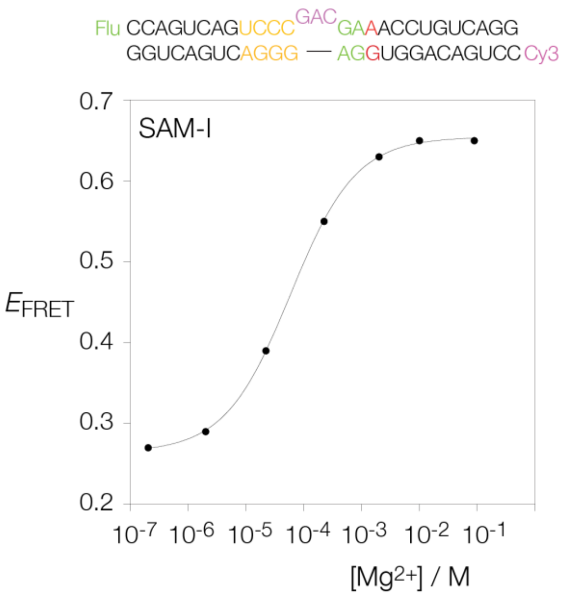

Supplementary Figure 2. The folding of the k-turn from the SAM-I riboswitch. This k-turn also folds fully in response to the addition of Mg^{2+} ions. The experimental data are shown by the filled circles, and the line is the fit to the data.

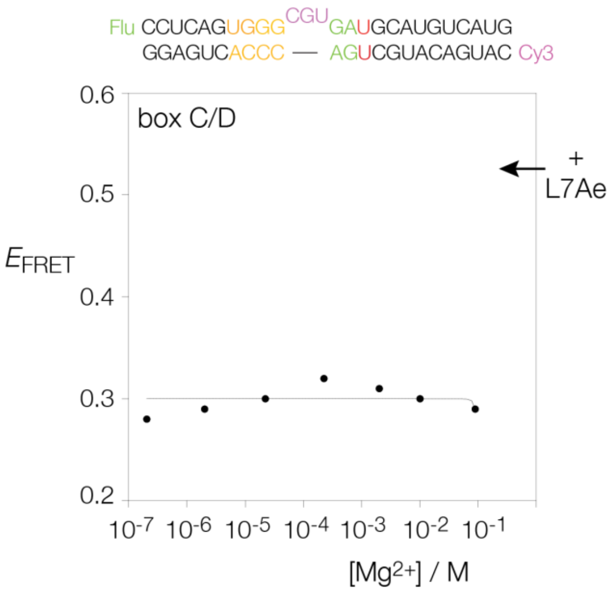

Supplementary Figure 3. Addition of Mg^{2+} ions to the box C/D k-turn brings about no change in FRET efficiency, i.e. no folding is induced. However, addition of the *Archeoglobus fulgidus* L7Ae protein leads to an $E_{FRET} = 0.51$ (arrow), i.e. the k-turn folds upon binding of the protein. The experimental data are shown by the filled circles, and the line is the fit to the data.

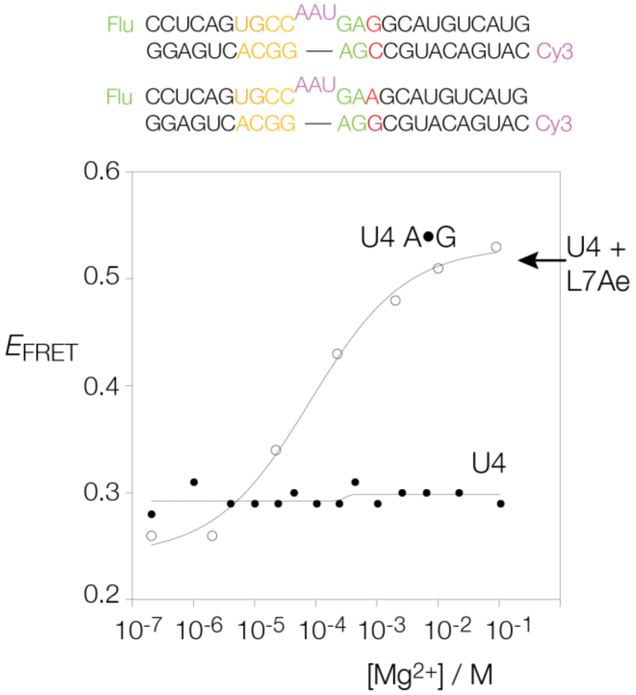

Supplementary Figure 4. Addition of Mg^{2+} ions to the human U4 snRNA k-turn brings about no change in FRET efficiency, i.e. no folding is induced. The experimental data are shown by the filled circles, and the line is the fit to the data. Addition of the *A. fulgidus* L7Ae protein leads to an $E_{FRET} = 0.53$ (arrow), i.e. the k-turn folds upon binding of the protein. E_{FRET} values for a variant of the U4 sequence in which the 3b•3n sequence was changed to G•A are also plotted as a function of Mg^{2+} ion concentration (open circles), and fitted to the two-state model (line). Note that this variant folds well in response to addition of Mg^{2+} ions.

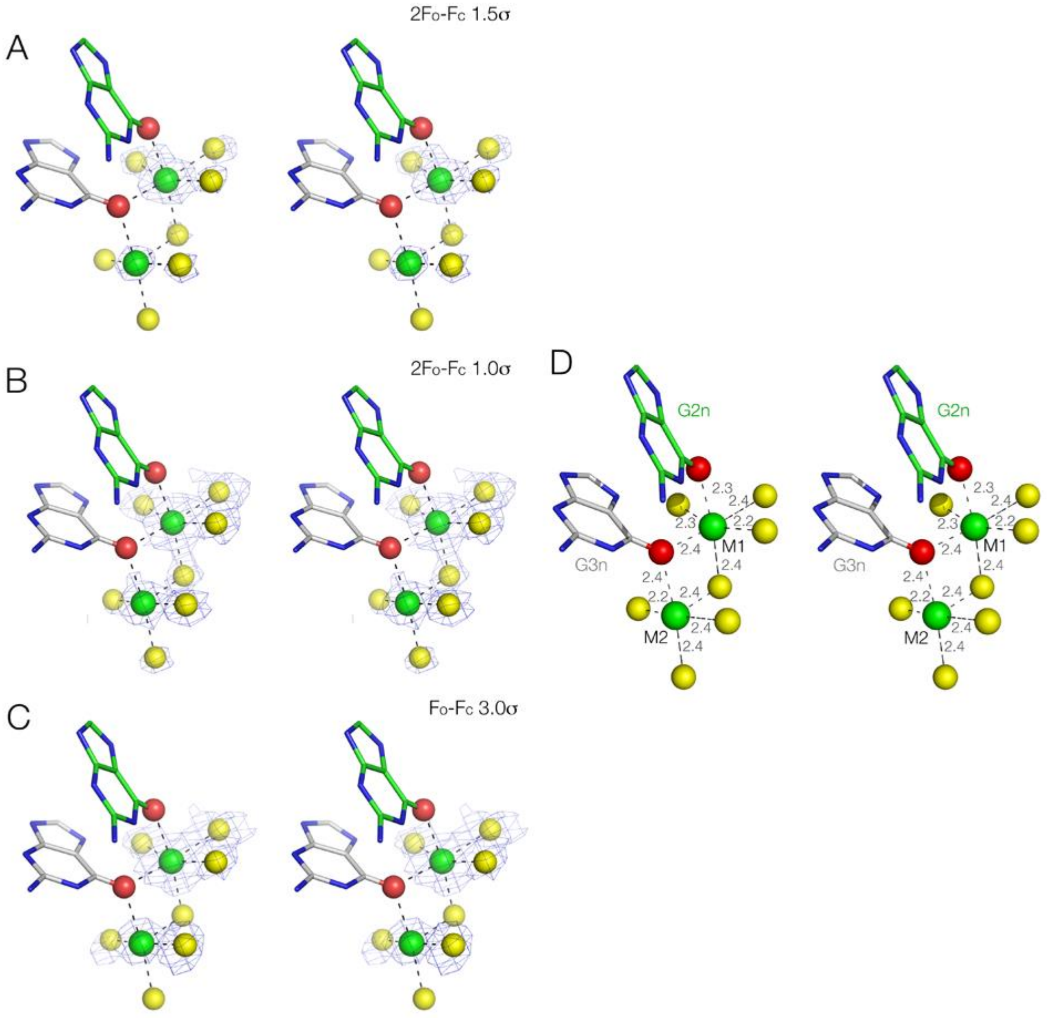

Supplementary Figure 5. The geometry and electron density maps of metal ions bound to the G2n and G3n positions of Kt-7. The metal ions are colored green and guanine O6 atoms red. Oxygen atoms of water molecules in the inner hydration spheres are colored yellow. The structures are shown in parallel-eye stereo.

A and **B**. 2$\mathbf{F_o}$-$\mathbf{F_c}$ electron density map contoured at 1.5σ and 1.0σ respectively.

C. $\mathbf{F_o}$-$\mathbf{F_c}$ electron density omit map contoured at 3.0σ.

D. The geometry of the bound metal ions with metal-oxygen interatomic distances shown (Å). The octahedral geometry and the metal-oxygen distances of 2.2 - 2.4 Å strongly suggests that the two metal ions are magnesium, but at this resolution we cannot exclude the possibility that they are sodium ions.

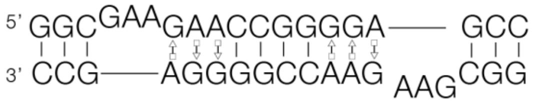

Supplementary Figure 6. The secondary structure of the k-turn-containing species that was crystallized.

Supplementary Table 1. Summary of k-turn folding data.

k-turn	Mg^{2+}	L7Ae
Kt-7 (AG)	0.56	0.58
SAM-I	0.65	0.57
box C/D	0.29	0.53
U4	0.35	0.54
U4 AG	0.53	
Kt-7 AA	0.24	
Kt-7 AC	0.34	
Kt-7 AU	0.21	0.60
Kt-7 GA	0.18	0.52
Kt-7 GG	0.51	
Kt-7 GC	0.17	0.58
Kt-7 GU	0.21	0.57
Kt-7 CA	0.54	
Kt-7 CG	0.17	
Kt-7 CC	0.56	0.59
Kt-7 CU	0.51	
Kt-7 UA	0.28	0.58
Kt-7 UG	0.51	
Kt-7 UC	0.16	0.57
Kt-7 UU	0.41	

Supplementary Table 1. Summary of final E_{FRET} values for all the k-turns studied in this work after titration of Mg^{2+} or addition of 40 nM *A. fulgidus* L7Ae protein. For the Kt-7 and U4 sequence variants, the 3b•3n sequence is shown.
